# Individual approach for treatment of primary intestinal lymphangiectasia in children: single-center experience and review of the literature

**DOI:** 10.1186/s12887-020-02447-5

**Published:** 2021-01-07

**Authors:** Yiyoung Kwon, Eun Sil Kim, Yon Ho Choe, Mi Jin Kim

**Affiliations:** Department of Pediatrics, Samsung Medical Center, Sungkyunkwan University School of Medicine, 81 Irwon-ro, Gangnam-gu, Seoul, 06351 South Korea

**Keywords:** Primary intestinal lymphangiectasia, Children, Individual approach, Review of the literature

## Abstract

**Background:**

Intestinal lymphangiectasia is a rare disease. Thus, prospective studies are impossible, and therapy is still controversial. Several medicines are suggested for treatment but there are no existing indications for drug choice and treatment guidelines. We aimed to introduce the action mechanism of each drug and treatment overview in a single-center experience and a review of the literature on second-line therapy for primary intestinal lymphangiectasia.

**Method:**

Children under 18 years old diagnosed with intestinal lymphangiectasia from June 2000 to June 2020 were included and retrospectively reviewed in the study. Capsule endoscopy, MR lymphangiography, or whole-body MRI for investigating the extent of abnormal lymphatic vessels in addition to endoscopy and biopsy were conducted. The individual treatment approaches depended upon the lymphangiectasis locations involved.

**Results:**

Only one patient showed a response to dietary therapy. One patient was successfully cured after two therapeutic lymphatic embolization. Octreotide was tried for two patients who had extensive lymphangiectasis. Lymphangiectasis recurred when octreotide was used for 3 months in one patient, and there was no effect in the other patient. Sirolimus was tried for four patients. Two of them had abnormal lymphatic lesions only in the intestine, and the others had extensive lymphangiectasis. The former group showed clinical improvement after 3–4 months of sirolimus treatment, whereas the latter group showed clinical improvement only after 1 month of sirolimus treatment.

**Conclusion:**

Surgery or embolization is a potential therapeutic option for patients with focal abnormal lymphatic lesions. Octreotide is not an optimal choice for patients with extensive lymphangiectasis. Sirolimus is an effective and safe drug and can be the first drug of choice for patients with extensive lymphangiectasis.

## Background

Intestinal lymphangiectasia is a rare disease that causes protein-losing enteropathy [1]. Clinical symptoms are induced by the excessive loss of lymphatic contents including protein, fat, and lymphocytes, resulting in hypoproteinemia and edema. Depending on the location of the injured lymphatic channel, pleural effusion, pericardial effusion, and ascites can develop as clinical features [1, 2].

All factors causing elevated lymph drainage pressure could lead to dilatation and even rupture of the lymphatic vessels [3]. Intestinal lymphangiectasia is classified into primary or secondary intestinal lymphangiectasia. In secondary intestinal lymphangiectasia, known factors trigger lymphatic channel injuries, such as heart surgery, chemotherapy, infection, or toxic substances [4]. Primary intestinal lymphangiectasia is also called idiopathic lymphangiectasia because of the absence of a known cause and is often congenital.

Since Waldmann reported the first case of intestinal lymphangiectasia in 1961, many case reports and articles have been published worldwide [3]. Nevertheless, treatment is still controversial, and prospective studies are not possible, especially since primary intestinal lymphangiectasia is a rare disease. Although the primary treatment strategy for secondary intestinal lymphangiectasia is to treat the underlying disease, there is no existing consensus on treatment for primary intestinal lymphangiectasia. To date, medicines like propranolol, octreotide, antiplasmin (e.g., tranexamic acid), and immunosuppressants (eculizumab and sirolimus) are suggested for treatment but there are no guidelines on indications for drug choice and treatment.

We experienced 18 pediatric patients with intestinal lymphangiectasia over 20 years. Among these patients, seven were diagnosed with primary intestinal lymphangiectasia. These seven patients showed clinical improvement with different treatment options. Our individual treatment approach depended upon the lymphangiectasis location involved. In this article, we aimed to introduce the action mechanism of each drug and an overview of our single-center treatment experience and a review of the literature on second-line therapy for primary intestinal lymphangiectasia.

## Methods

### Patient characteristics and diagnosis

Children under 18 years old diagnosed with intestinal lymphangiectasia from June 2000 to June 2020 in the Department of Pediatric Gastroenterology and Nutrition were evaluated retrospectively. We included patients who were followed-up for more than 2 years. The total number of patients initially diagnosed with intestinal lymphangiectasia was 18, but excluded 9 patients with secondary intestinal lymphangiectasia. Two patients were also excluded because they were lost to follow-up. Thus, seven patients were included in this study.

The diagnosis was based on typical endoscopic small bowel findings and confirmed by histology. Endoscopic findings like a snowflake appearance, diffusely prominent white villi, and small whitish patches were taken as suggestive features of intestinal lymphangiectasia [5]. Two or three biopsy segments were taken from the second or third part of the duodenum and histological analysis revealed dilated lymphatic channels in the lamina propria.

The extent of the lymphangiectatic area was determined by capsule endoscopy, MR lymphangiography or whole-body MRI [6, 7]. As a diagnostic process, serum albumin and globulin levels and lymphocyte counts were checked, and alpha-1 antitrypsin level in stool was evaluated [8].

**Fig. 1:**
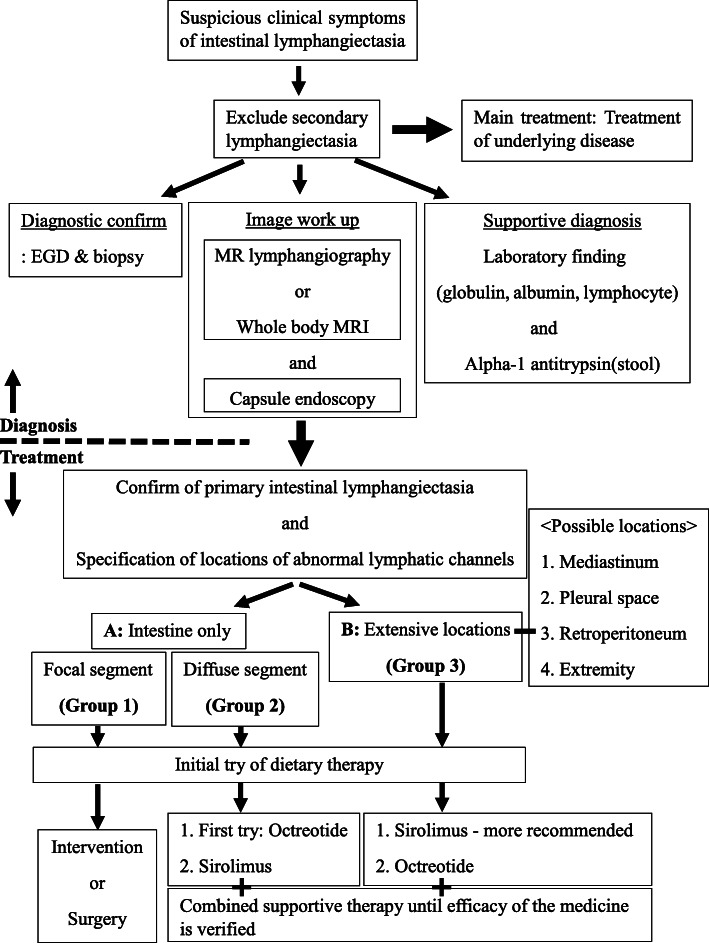
Diagnostic flow and individual therapeutic strategy of primary intestinal lymphangiectasia

We divided the patients into three groups for treatment. Group 1 is focal intestinal lymphangiectasia group. Group 2 is diffuse intestinal lymphangiectasia group. Lastly, group 3 is extensive-type lymphangiectasia group (extraintestinal involvement such as pleural space, mediastinum, retroperitoneum, extremities) (Fig. 1).

### Treatment and follow-up

All patients were initially managed with supportive therapies, such as correcting electrolyte imbalances, replacing albumin, and using diuretics. Some patients required paracentesis or thoracentesis because of effusion and total parenteral nutrition (TPN). After an improvement in their general condition, all patients were routinely supported with dietary therapy composed of high protein and medium-chain triglycerides, but not very low long-chain triglyceride. Calcium and fat-soluble vitamins were also supplemented [9].

Patients who did not show clinical improvement after 1–2 months of dietary therapy were considered for second-line therapy. Second-line therapy referred to lymphangiectasis treatment after the initial treatment (the first-line treatment was dietary therapy in this study) failed. The options for second-line therapy were surgery or radiologic intervention for focal-type lymphangiectasia, or medications like octreotide and sirolimus for the extensive-type of lymphangiectasia.

There are no standardized recommended doses or duration of octreotide therapy. As an induction therapy, 1–10 mcg/kg/dose was injected subcutaneously twice a day for 2 weeks, and the same dose was injected subcutaneously at 4-week intervals after induction.

Sirolimus was administered orally on a continuous dosing schedule at a starting dose of 2 mg daily. The drug trough level was checked regularly, twice weekly and maintained between 5 to 15 ng/mL [10]. We monitored adverse effects like cytopenia, tachycardia, hepatotoxicity, hyperglycemia, and electrolyte imbalance.

After discharge, all patients were followed-up regularly in the outpatient department with physical examinations, evaluation of growth, and blood tests.

## Results

The clinical features of seven patients with primary intestinal lymphangiectasia are described in Table 1. All patients were hospitalized at diagnosis. The initial treatment for all patients were MCT enriched high protein content diet. Only Patient 1 responded to diet therapy and the therapeutic effect, which started after 1 week, showed improvement not only in the serum albumin level (from 1.8 g/dL to 2.7 g/dL) but also in clinical symptoms such as diarrhea and anasarca. The other six patients, who were put on a strict diet, seemed to have clinical improvement in about a week but no significant increase in albumin level. After discharge, the symptoms resumed due to incompliance to the dietary treatment. Patients who developed refractory hypoalbuminemia, increased generalized edema and ascites despite dietary reeducation and conservative treatment for 1–3 months were considered for second-line therapies.

**Table 1:** Clinical characteristics of 7 patients diagnosed with primary intestinal lymphangiectasia. wks; week-old, yr.; year-old

Patient number	Age range at diagnosis^a^	Time interval of 2nd-line therapy^b^	Group	Type of lymphangiectasia	Intestinal involve	3rd space involved	Response to diet therapy	Type of 2nd-line therapy
1	Infant	–	1	Focal intestine	Focal	No	Yes	None
2	Child	0.5 yr	1	Focal intestine	Focal	No	No	Surgery
3	Child	8 yr	1	Focal intestine	Focal	No	No	Embolization
4	Adolescent	0.7 yr	3	Extensive	Diffuse	Yes	No	Octreotide➔Sirolimus
5	Neonate	3 yr	3	Extensive	Diffuse	Yes	No	Octreotide➔Sirolimus
6	Neonate	8.9 yr	2	Diffuse intestine	Diffuse	No	No	Sirolimus
7	Child	0.7 yr	2	Diffuse intestine	Diffuse	No	No	Sirolimus

^a^There is an accurate diagnosis age, but age is expressed as a range for anonymity

^b^The time when the second line therapy started from the diagnosis

When we chose second-line therapy, we primarily considered two main factors, the location and extent of abnormal lymphatic channels. We had information on these factors because of initial evaluations conducted with capsule endoscopy, whole-body MRI, or MR lymphangiography.

Patient 2 had abnormal lymphatic lesions only in the small intestine (Group 1), but the lesions were slightly broad. Small bowel resection was planned for his lymphangiectasis. Resection from 70 cm below the Treitz ligament to 130 cm above the IC valve was completed successfully without complications. The albumin level, which was initially 2.0 g/dL. was elevated to 4.4 g/dL after surgery.

**Fig. 2:**
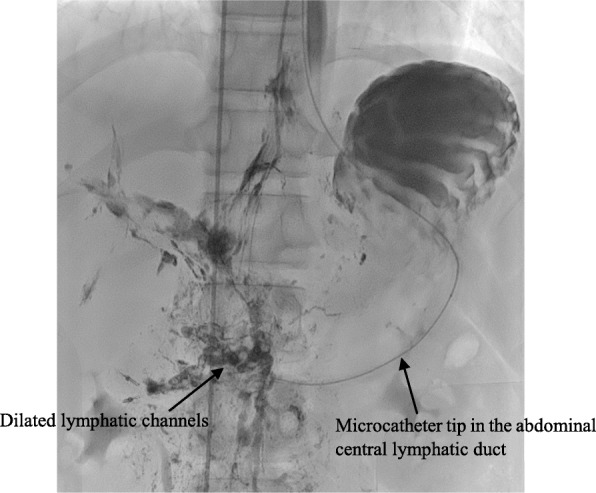
Nodal lymphangiography and therapeutic embolization is performed using diluted glue in Patient 3

Patient 3 had abnormal lymphatic channels only in the duodenal lesion (Group 1). Although the extent of the lesion was small, pylorus-preserving pancreaticoduodenectomy was considered as a surgical method due to its location. After a multidisciplinary discussion, we decided to attempt lymphatic embolization instead of surgery. The patient’s albumin level, which was initially 1.5 g/dL, was elevated to 4.9 g/dL after two lymphatic embolization (Fig. 2).

**Fig. 3:**
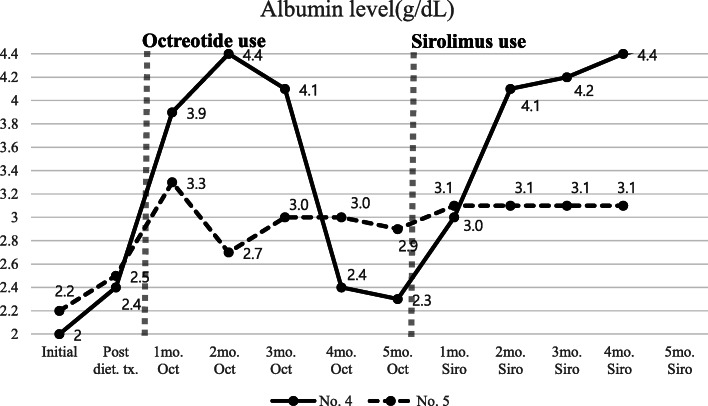
Serum albumin level trends of patients who are treated with sirolimus after octreotide failure. Diet.; dietary, mo.; month, Oct.; Octreotide, Siro.; Sirolimus, No. 4; Patient 4, No. 5; Patient 5

Patients 4 (Group 3) and 5 (Group 3) had extensive abnormal lymphatic lesions in their bodies. The lesions not only involved broad intestinal segments but also lymphatic channels in the mediastinum and retroperitoneum. Even the extremities in Patient 5 were involved. They had clinical symptoms of ascites, generalized edema and pleural effusions. With the use of diuretics as a supportive therapy, the patients were treated with octreotide. Patient 4 was maintained with octreotide for 1 year. Octreotide seemed to be effective for 3 months, but the patient’s symptoms started to aggravate again without any specific event and the drug was discontinued. Patient 5 underwent octreotide induction therapy three times, but there was no efficacy at all. After repeated hospitalization, we decided to use another trial of sirolimus treatment. Clinical improvement emerged in both patients after 1 month of sirolimus use (Fig. 3). In the case of Patient 4, sirolimus treatment was maintained for 3 years. She presented with clinical remission (no lymphangiectasis symptoms) after treatment with sirolimus for about 2 years. In the case of Patient 5 who had presented with severely refractory lymphangiectasis, his albumin level was still low at 3.1 g/dL with sirolimus use, but he no longer needed paracentesis or thoracentesis. He was treated with sirolimus only for 4 months, and we are still following him in the outpatient clinic.

**Fig. 4:**
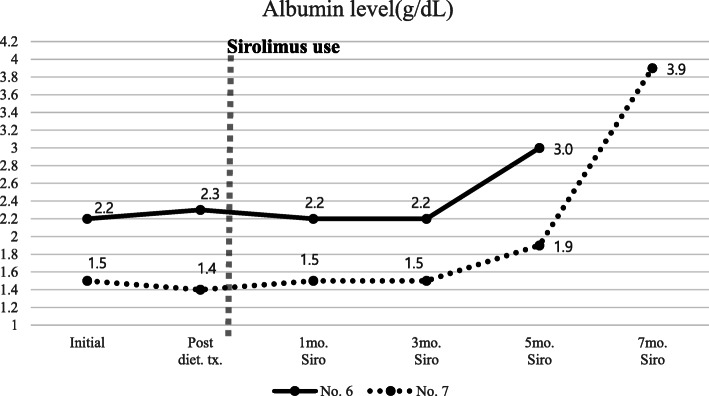
Serum albumin level trends of each patient who are treated with sirolimus as secondary medication therapy. Diet.; dietary, mo.; month, Siro.; Sirolimus, No. 6; Patient 6, No. 7; Patient 7

Patient 6 and 7 have been recently diagnosed and any surgery or intervention have not been decided yet. They also had broad abnormal lymphatic lesions in the intestine, duodenum, and all sections of the small intestine. Therefore, we decided to start medication therapy and chose sirolimus, not octreotide, based on our prior experience with octreotide treatment failure in extensive-type lymphangiectasis. We regularly checked the sirolimus trough and albumin levels and clinical symptoms. The albumin levels started to increase after 3 months of sirolimus use and the symptoms slowly relieved (Fig. 4).

## Discussion

The basic treatment for primary intestinal lymphangiectasia is dietary therapy. We tried dietary therapy as an initial treatment in all patients, but since dietary therapy was not effective in most of the patients, we became to try various second-line treatments. We made a suggestive treatment guide by evaluating which treatment was effective in patients through classification according to the extent of the lesion and action mechanisms of drugs.

Dietary therapy for our patients consisted of high protein and low fat substituted with MCTs. The exclusion of long-chain fatty acids reduces lymphatic flow and pressure, and thus prevents the rupture of malformed lymphatics, while MCTs are directly absorbed into the portal venous circulation and bypass the enteric lymphatics [11]. Several case reports and articles have introduced the long-term effect of dietary therapy [12–19]. One patient in our study responded to dietary therapy (Table 1) and maintained clinical improvement for more than 5 years.

Having response to dietary therapy is ideal but some patients are non-responsive to dietary therapy. For that reason, more than 10 reports have introduced secondary therapy like surgery, octreotide, or sirolimus, but there is no consensus on how to choose and apply these therapies to patients who are refractory to dietary therapy [2, 20–32]. We tried to suggest a reasonable choice of second-line therapy because we had several experiences with therapeutic challenges and success with multimodal treatment options. Although no discussion has been made in existing papers on when to start secondary treatment, we think it is sufficient to evaluate the response with 2 weeks of dietary therapy, given our experience in treatment and other papers on dietary reactions [33].

We emphasize that the initial evaluation of the location and the extra-intestinal extent of abnormal lymphatic lesions is important for a therapeutic strategy. We divided our patients into three groups to distinguish and assess the efficacy of the second-line treatments. We also analyzed other published cases using the same protocol and conducted a review of the literature on second-line therapy (Table 2).

**Table 2:** Summary of articles/case reports of intestinal lymphangiectasia treated with second-line therapy. wks; week-old, yr.; year-old, mo.; month-old

Author	Age	Group	Type of lymphangiectasia	Intestinal involve	3rd space involve	Response to diet therapy	Type of 2nd-line therapy	Results of 2nd-line therapy
CP Chen et al. (2003) [24]	49 yr.	1	Focal intestine	Focal	No	No	Surgical resection	Remission
L Zhu et al. (2010) [32]	22 yr.	1	Focal intestine	Focal	No	Limited data	Surgical resection	Remission
	44 yr.	1	Focal intestine	Focal	No	Limited data	Surgical resection	Remission
	71 yr.	1	Focal intestine	Focal	No	Limited data	Surgical resection	Remission
	55 yr.	1	Focal intestine	Focal	No	Limited data	Surgical resection	Remission
W Kneist et al. (2013) [25]	58 yr.	1	Focal intestine	Focal	No	No	Surgical resection	Remission
J Mari et al. (2019) [28]	10 yr.	1	Focal intestine	Focal	No	Limited data	Surgical resection	Remission
G Kuroiwa et al. (2001) [26]	21 yr.	1	Focal intestine	Focal	No	No	Octreotide	Recurred after discontinuation
L Filk et al. (2004) [34]	25 yr	Limited data	Limited data	Limited data	Limited data	No	Octreotide	Clinical improvement
S Sari et al. (2010) [30]	*N* = 6, age 2–24 mo.	Limited data	Limited data	Limited data	Limited data	No	Octreotide	Clinical improvement
Suehiro et al. (2012) [22]	63 yr.	1	Focal intestine	Focal	No	No	Octreotide	Clinical improvement
MJ Alshikho et al.(2016) [35]	24 yr	Limited data	Limited data	Limited data	Limited data	No	Coconut oil + Octreotide	Clinical improvement
Troskot et al. (2015) [31]	42 yr.	2	Diffuse intestine	Diffuse	No	No	Octreotide	Clinical improvement
Z Altın et al. (2018) [2]	34 yr.	3	Extensive	limited	Yes	No	Octreotide	Recurred after discontinuation
Acer-Demir et al. (2020) [23]	3 yr.	3	Extensive	Diffuse	Yes	No	Octreotide ➔ Surgical resection	Died d/t uncontrolled symptoms
MacLean et al. (2002) [27]	14 yr.	3	Extensive	Diffuse	Yes	No	Octreotide➔ Tranexamic acid	Clinical improvement
D Prasad et al. (2019) [29]	*N* = 2, age 2–18 yr.	2	Diffuse intestine	Diffuse	No	No	Octreotide	Clinical improvement
	*N* = 4, age 1–10 yr.	3	Extensive	Diffuse	Yes	No	Octreotide + Tranexamic acid	Clinical improvement
Ozeki et al. (2016) [20]	12 yr.	3	Extensive	Diffuse	Yes	No	Propranolol➔Everolimus	Remission for 12 months

We evaluated capsule endoscopy and MRI after imaging modality has been developed in addition to esophagogastroduodenoscopy. After confirming the locations of the abnormal lymphatic lesions, we considered surgery after dietary therapy failure for the patients with focal abnormal lesions because surgery is the only treatment option with a chance of a complete cure. Cases reporting surgical treatment for patients with focally affected lesions have been published, and all patients showed clinical remission, consistent with our case (Table 2).

With the development of radiologic intervention, we attempted lymphatic embolization instead of surgery in one patient and the result was very successful. This was the first case of recovery from primary intestinal lymphangiectasia and clinical remission in a child or an adult treated with embolization. This procedure minimizes the risk of complications and decreases the treatment period commonly associated with surgery [36–38]. Embolization is a potential therapeutic option for focal lesions of primary intestinal lymphangiectasia in children. Surgery can be considered after embolization treatment failure.

For the patients whose disease extent is too broad to undergo embolization or surgery, medical therapy should be considered for second-line medical therapy. We have treatment experience with octreotide and sirolimus. Choosing the appropriate drug for patients is challenging for all clinicians.

Octreotide is a somatostatin analog whose mechanisms include decreased intestinal absorption of fats, inhibition of gastrointestinal vasoactive peptides, and stimulation of the autonomic nervous system [22]. Because of its mechanisms, we hypothesized that it is optimal for patients who have only intestinal involvement of the abnormal lymphatics with severe diarrhea, but we only have experience using the drug with extensive-type lymphangiectasis. Octreotide had little therapeutic effect in these patients. Ten case reports presenting experience treating with octreotide have been published. Consistent with our experience, octreotide showed little effect in patients with extensive abnormal lymphatic lesions. There are also reports that describe the recurrence of lymphangiectasis after the discontinuation of octreotide. Sari et al. (2010) [30], Prasad et al. (2019) [29], and some other reports described clinical improvement after octreotide treatment but there is no information on the long-term efficacy of octreotide (Table 2) [34, 35]. We analyzed patient characteristics in the aspects of location and extent of lymphangiectasis in reports of clinical outcomes after using octreotide. Like our prediction, patients in reports who have lymphangiectasis only in intestine, responded to octreotide treatment without recurrence after discontinuation. Otherwise, patients in reports who have lymphangiectasis extensively, failed to octreotide treatment (Table 2). In that reason, we made an attempt to use sirolimus initially in patient with extensive lymphangiectasis.

Sirolimus acts on lymphatic endothelial cells and changes mTOR signaling, suppressing lymphatic sprouting and proliferation, and inducing apoptosis [39]. We tried sirolimus in four patients (Patients 4, 5, 6, and 7) who failed to respond to dietary therapy initially and two of them also failed to respond to octreotide therapy (Table 1). Among the four patients, two patients (Patients 6 and 7) with abnormal lymphatic lesions only in the intestine showed clinical improvement after 3–4 months of sirolimus treatment, whereas the other two patients (Patients 4 and 5) who had extensive-type lymphangiectasis showed clinical improvement only after on month of sirolimus treatment (Figs. 3 and 4). The way sirolimus acts on lymphatic channels resulted in different effect onset times in these two groups. Because sirolimus acts on endothelial cells of the lymphatic channel, not by controlling lymphatic flow like octreotide or dietary therapy, it can affect any lymphatic vessels in the body. We concluded that patients who had the extensive form of abnormal lymphatics channels could be initially considered for sirolimus treatment rather than octreotide for a fast response and cure (Fig. 1). One case report of treatment experience with everolimus (an mTOR inhibitor drugs) was published in Japan. Everolimus was prescribed because it was not possible to use sirolimus in the hospital. Their patient characteristics were similar to our patients who had extensive abnormal lymphatic lesions in the body (Table 2). The drug effect was seen after 4 weeks of use, like in our cases. However, the appropriate drug discontinuation time is debatable because there is no current consensus or guidelines [40, 41]. Incidence of many adverse effects of sirolimus is dose related, and a frequent adverse effect of sirolimus, which is up to 40%, is elevation of creatinine level. Everolimus has an advantage over sirolimus in preserving kidney function, because it is metabolized in the liver via CYP3A4. Another troublesome adverse effect of sirolimus is lymphedema mainly in extremities. There are reports of lymphedema following the use of sirolimus after kidney or liver transplantation. The reported median time between lymphedema onset and the beginning of sirolimus was 52 weeks [42]. Therefore, by this time, the drug concentration should be checked periodically and carefully monitored for side effects.

Only two case reports presented treatment experience with tranexamic acid [27, 29]. Tranexamic acid 25 mg/kg/dose three times a day was used orally (maximum 1000 mg) for 5 days [29], and patients showed clinical improvement after 1 month of treatment [27]. The mechanism of antiplasmin therapy is the normalization of tissue fibrinolytic activity [43]. Increased fibrinolytic activity, which causes intestinal protein loss, has been proposed as a mechanism. Mine et al. [44] suggested that there is a subset of patients with lymphangiectasia who may have increased tissue or plasma fibrinolytic activity and may respond to antiplasmin therapy. Elevated D-dimers may reflect increased fibrinolytic activity. Tranexamic acid can be a choice for patients who present with refractory symptoms of lymphangiectasis.

The primary limitation of our study was its retrospective nature design and the small number of patients. It is practically impossible to perform prospective studies because primary intestinal lymphangiectasia is a very rare disease. We also need more follow-up information after the discontinuation of sirolimus.

## Conclusion

Interventional treatment is a potential therapeutic option for patients with focal abnormal lymphatic lesions. Octreotide can be tried for patients with abnormal lymphangiectasis only in the intestine, with symptoms of diarrhea. However, it is not optimal for patients with extensive lymphangiectasis. Sirolimus is an effective drug for patients with extensive lymphangiectasis and a safe drug even for young pediatric patients.

Our future challenge is formulating recommendations for ideal treatment periods and the timing of sirolimus discontinuation. An individual therapeutic approach after an objective diagnostic evaluation improves the chances of disease remission.

## Data Availability

The datasets used and analyzed during the current study are available from the corresponding author on reasonable request.

## Abbreviations

MR: Magnetic resonance
MRI: Magnetic resonance imaging
No.: Number
MCT: Medium chain triglyceride
mTOR: Mammalian target of rapamycin

## References

[1] Vignes S, Bellanger J (2008). Primary intestinal lymphangiectasia (Waldmann’s disease). Orphanet J Rare Dis.

[2] Altın Z (2018). Primary intestinal lymphangiectasia and a review of the current literature. Turk J Gastroenterol.

[3] Lee J, Kong M-S (2008). Primary intestinal lymphangiectasia diagnosed by endoscopy following the intake of a high-fat meal. Eur J Pediatr.

[4] Wilkinson P, Pinto B, Senior JR (1965). Reversible protein-losing enteropathy with intestinal lymphangiectasia secondary to chronic constrictive pericarditis. N Engl J Med.

[5] Asakura H (1981). Endoscopic and histopathological study on primary and secondary intestinal lymphangiectasia. Dig Dis Sci.

[6] Lee EW (2014). Lymphangiography to treat postoperative lymphatic leakage: a technical review. Korean J Radiol.

[7] Rivet C (2006). Use of capsule endoscopy in children with primary intestinal lymphangiectasia. Gastrointest Endosc.

[8] Bernier J (1978). Diagnosis of protein-losing enteropathy by gastrointestinal clearance of alpha1-antitrypsin. Lancet.

[9] Jeffries GH, Chapman A, Sleisenger MH (1964). Low-fat diet in intestinal lymphangiectasia: its effect on albumin metabolism. N Engl J Med.

[10] Ingle GR, Sievers TM, Holt CD (2000). Sirolimus: continuing the evolution of transplant immunosuppression. Ann Pharmacother.

[11] Suresh N (2009). Primary intestinal lymphangiectasia. Indian Pediatr.

[12] Aroor S (2017). Waldmann’s disease (primary intestinal Lymphangiectasia) with atrial Septal defect. J Clin Diagn Res.

[13] Desai A, Guvenc B, Carachi R (2009). Evidence for medium chain triglycerides in the treatment of primary intestinal lymphangiectasia. Eur J Pediatr Surg.

[14] Isa HM, Al-Arayedh GG, Mohamed AM (2016). Intestinal lymphangiectasia in children: a favorable response to dietary modifications. Saudi Med J.

[15] Mohammad L (2019). Primary intestinal lymphangiectasia in a 23-month-old girl. Oxford Med Case Rep.

[16] Surampalli V (2017). Primary intestinal Lymphangiectasia (Waldmann’s disease) presenting with Chylous effusions in a 15-year-old. J Clin Diagn Res.

[17] Wang X, Jin H, Wu W (2016). Primary intestinal lymphangiectasia manifested as unusual edemas and effusions: a case report. Medicine.

[18] Tang Q-Y (2011). Clinical outcome of nutrition-oriented intervention for primary intestinal lymphangiectasia. World J Pediatr.

[19] Tift W, Lloyd J (1975). Intestinal lymphangiectasia. Long-term results with MCT diet. Arch Dis Child.

[20] Ozeki M (2016). Everolimus for primary intestinal lymphangiectasia with protein-losing enteropathy. Pediatrics.

[21] Strauss J, Gidrewicz D, McKenzie L (2019). A148 Sirolimus for primary intestinal lymphangiectasia in a pediatric patient. J Canadian Assoc Gastroenterol.

[22] Suehiro K (2012). Late-onset primary intestinal lymphangiectasia successfully managed with octreotide: a case report. Ann Vasc Dis.

[23] Acer-Demir T, Ötgün I, Özçay F (2020). First report of treatment with pancreas-sparing duodenectomy in a child with primary intestinal lymphangiectasia. J Indian Assoc Pediatr Surg.

[24] Chen C-P (2003). Surgical resection of duodenal lymphangiectasia: a case report. World J Gastroenterol: WJG.

[25] Kneist W (2013). Surgical therapy of segmental jejunal, primary intestinal lymphangiectasia. Zeitschrift fur Gastroenterologie.

[26] Kuroiwa G (2001). Primary intestinal lymphangiectasia successfully treated with octreotide. J Gastroenterol.

[27] MacLean JE, Cohen E, Weinstein M (2002). Primary intestinal and thoracic lymphangiectasia: a response to antiplasmin therapy. Pediatrics.

[28] Mari J (2019). Pediatric localized intestinal lymphangiectasia treated with resection. Int Med Case Rep J.

[29] Prasad D (2019). Clinical profile, response to therapy, and outcome of children with primary intestinal lymphangiectasia. Dig Dis.

[30] Sari S, Baris Z, Dalgic B (2010). Primary intestinal lymphangiectasia in children: is octreotide an effective and safe option in the treatment?. J Pediatr Gastroenterol Nutr.

[31] Troskot R (2015). How to treat an extensive form of primary intestinal lymphangiectasia?. World J Gastroenterol: WJG.

[32] Zhu L-h (2010). Partial enterectomy: treatment for primary intestinal lymphangiectasia in four cases. Chin Med J.

[33] Li R (2015). Dietary or enteral medium-chain triglyceride usage in a Chinese general hospital. Asia Pac J Clin Nutr.

[34] Filik L (2004). A case with intestinal lymphangiectasia successfully treated with slow-release octreotide. Dig Liver Dis.

[35] Alshikho MJ (2016). Intestinal lymphangiectasia: insights on management and literature review. Am J Case Rep.

[36] Kawasaki R (2013). Therapeutic effectiveness of diagnostic lymphangiography for refractory postoperative chylothorax and chylous ascites: correlation with radiologic findings and preceding medical treatment. Am J Roentgenol.

[37] Nadolski GJ, Itkin M (2012). Feasibility of ultrasound-guided intranodal lymphangiogram for thoracic duct embolization. J Vasc Interv Radiol.

[38] Rajebi MR (2011). Intranodal lymphangiography: feasibility and preliminary experience in children. J Vasc Interv Radiol.

[39] Baluk P (2017). Rapamycin reversal of VEGF-C–driven lymphatic anomalies in the respiratory tract. JCI insight.

[40] Hu S (2019). Long-term efficacy and safety of sirolimus therapy in patients with lymphangioleiomyomatosis. Orphanet J Rare Dis.

[41] Wiegand S, Wichmann G, Dietz A (2018). Treatment of lymphatic malformations with the mTOR inhibitor sirolimus: a systematic review. Lymphat Res Biol.

[42] Fourgeaud C (2018). Lymphedema in patients treated with sirolimus: 15 cases. La Revue de medecine interne.

[43] Kondo M (1976). Experimental protein-losing gastroenteropathy: role of tissue plasminogen activator. Gastroenterology.

[44] Mine K (1989). Intestinal lymphangiectasia markedly improved with antiplasmin therapy. Gastroenterology.

